# Response of microbial community structure and metabolic profile to shifts of inlet VOCs in a gas-phase biofilter

**DOI:** 10.1186/s13568-018-0687-z

**Published:** 2018-10-03

**Authors:** Lichao Lu, Guangchun Wang, Marvin Yeung, Jinying Xi, Hong-Ying Hu

**Affiliations:** 1 Environmental Simulation and Pollution Control State Key Joint Laboratory, School of Environment, Tsinghua University, Beijing, China; 2 Graduate School at Shenzhen, Tsinghua University, Shenzhen, China; 3 Central Research Institute of Building and Construction, MCC Group, Co, Ltd., Beijing, China

**Keywords:** VOCs, Biofiltration, Microbial community, Metabolic profile

## Abstract

The effects of inlet VOCs (Volatile Organic Compounds) shifts on microbial community structure in a biofiltration system were investigated. A lab-scale biofilter was set up to treat eight VOCs sequentially. Short declines in removal efficiency appeared after VOCs shifts and then later recovered. The number of OTUs in the biofilter declined from 690 to 312 over time. At the phylum level, *Actinobacteria* and *Proteobacteria* remained dominant throughout the operation for all VOCs, with their combined abundance ranging from 60 to 90%. The abundances of *Planctomycetes* and *Thermi* increased significantly to 20% and 5%, respectively, with the intake of non-aromatic hydrocarbons. At the genus level, *Rhodococcus* was present in the highest abundance (≥ 10%) throughout the experiment, indicating its wide degradability. Some potential degraders were also found; namely, *Thauera* and *Pseudomonas*, which increased in abundance to 19% and 12% during treatment with ethyl acetate and toluene, respectively. Moreover, the microbial metabolic activity declined gradually with time, and the metabolic profile of the toluene-treating community differed significantly from those of other communities.

**Electronic supplementary material:**

The online version of this article (10.1186/s13568-018-0687-z) contains supplementary material, which is available to authorized users.

## Introduction

There has been growing interest in control of the emission of volatile organic compounds (VOCs) because of their potential to harm the environment and human health toxicity (Zheng et al. 2013). Recently, waste gases emitted in high volume containing complex VOCs at low concentration have hindered performance enhancement of related treatments (Cheng et al. 2016). This has led to increased interest in biofiltration systems, which have advantages of high efficiency, minimal secondary pollution and low costs (Khan and Ghoshal 2000).

Microorganisms play an important role in biodegradation, having direct interactions with contaminants. In previous studies, significant shifts in the bacterial community were observed during biodegradation, especially in the initial period (Qiu et al. 2013). Increasing doses also impact microbial communities (Li et al. 2017). Efforts were also paid on microbial community structures analysis to determine microbial indicators of contaminants (Obi et al. 2017). Evaluation of the influence of different degrading conditions on microbial communities also revealed significant effects (Techtmann et al. 2017). However, it is worth noting that previous studies were predominantly conducted using simple and stable inlet chemicals, while few investigations have focused on microbial changes with contaminant shifts.

Various VOCs have physical and chemical characteristics that differ significantly. Water-solubility and biodegradability are the two main factors that influence the removal efficiency of biofilters (Deshusses and Webster 2000). Low water-solubility limits mass transfer prior degradation (Alonso et al. 1998), while biodegradability influences the degradation of soluble VOCs. Among all pollutants, hydrocarbons (i.e., alcohols, ketones, esters etc.) have been shown to be the easiest degraded compounds, before alkene and aromatic hydrocarbon (Delhomenie and Heitz 2005). Therefore, different types of VOCs alter biofilter performances and microbial community structures significantly. However, precise analyses and conclusive studies are not yet available, especially for waste air treatments.

Few studies have investigated the interactions between environments and microorganisms (Zhang et al. 2018); therefore, the present study was conducted to elucidate these interactions. Specifically, this study investigated a biofilter applied to treat a variety of volatile organic compounds (toluene, ethylbenzene, chlorobenzene, acetone, isopropyl alcohol, ethyl acetate, n-hexane and tetrahydrofuran) in sequence. Differences in degrading performance and the changes in microbial structures during the process were then evaluated. The data obtained in this study will provide insight into microbial community function, functional diversity, and other aspects of the biofilter operation.

## Materials and methods

### Biofilter configuration and operation

#### Biofilter set up

The system had a height of 335 mm and an internal diameter of 118, giving an effective height of packing material of 150 mm with an approximately 1.7 L volume (Additional file 1: Figure S1). The air flow was pressurized and controlled with an electromagnetic air compressor (ACO-318, Hailea Co., Ltd., Guangdong, China) and a flowmeter (LZB-WB, Zhenxing Flowmeter Factory, China). Pressurized airflow entered the mixing chamber, which contained multiple vials of evaporating VOCs. The quantity of the vials, volume and volatile area of the VOCs was used to control the inlet concentration. Mixed air flow then entered the biofilter from the bottom and passed through the packing materials containing the microorganisms, after which the treated air was discharged from the top of the biofilter. An electromagnetic flowmeter (Iwaki Co., Ltd, EH-B20VC-220R1) was used to control the liquid flow rate and a microcomputer time controlled switch (Toone Co., Ltd., Shanghai, China) was used to control the spraying rate.

Eight common volatile organic compounds were selected in this study for biofilter performances and microbial community analyses. The selected compounds, including aromatic and non-aromatic hydrocarbons, were common industrial materials or organic solvents (Additional file 1: Table S1). Different VOCs were applied one by one in the order toluene, ethylbenzene, chlorobenzene, acetone, isopropyl alcohol, ethyl acetate, *n*-hexane and tetrahydrofuran. The duration of operation for each compound ranged from 12 to 17 days, depending on the time spent to reach a stable state.

#### Biofilter operation

The inlet air flow rate was 2.2 L min^−1^ with an empty bed residence time of 46 s. Spraying was conducted for 1 min every 3 h, giving a total sprayed volume of 90 mL. The nutrient medium was renewed every 3–4 days, the pH of the medium was controlled at 6.5–7.2, and temperature ranged from 25 to 32 °C.

#### Nutrient medium and inoculation

The nutrient medium used consisted of 10 g NaNO_3_, 2.56 g Na_2_HPO_3_ and 1.66 g KH_2_PO_3_ per L water, and the pH values ranged from 6.5 to 7.2. The medium was inoculated with suspended activated sludge, and 1.7 L packing materials were filled into the biofilter after being soaked in 1 L of the activated sludge suspension for an hour.

### Analytical methods

#### VOCs, CO_2_ and biomass concentration

The concentrations of the chemicals were analyzed by gas chromatography (GC-14C, Shimadzu Co. Ltd., Shanghai, China). The temperatures of the inlet, column (HICRON HP-1, 50 m × 0.25 mm) and detector were 150 °C, 100 °C and 150 °C, respectively.

The concentration of CO_2_ was tested using a portable CO_2_ m (Testo 535, Testo China Co. Ltd., China), while decreases in pressure were analyzed with a manometer (Testo 512, Testo China Co. Ltd., China).

Biomass in different periods was tested 2 h after spraying to minimize the influence of sprayed nutrient medium. The growth of biomass was compared among treatments to evaluate increases in microorganisms.

#### DNA extraction

DNA extraction was performed during the stable phase using a Fast DNA™ SPIN Kit for Soil (MP Biomedicals, Canada), after which the extracted DNA was sequenced using the Illumina MiSeq sequencing platform (Novogene Co., Beijing, China) and the 515F (5′-GTGCCAGCAGCCGCGGTAA-3′) and 806R (5′-GGACTACCAGGGTATCTAAT-3′) targeting the V4 region of the 16S rRNA gene. Each reverse primer contained a 6-bp error-correcting unique barcode and PCR amplification was conducted by subjecting the samples to 98 °C for 5 min, followed by 40 cycles of 94 °C for 30 s, 55 °C for 30 s and 72 °C for 45 s, and then final extension at 72 °C for 10 min. Sequences were then analyzed using the Illumina MiSeq platform (Novogene Co., Beijing, China).

#### DNA sequencing

Illumina MiSeq original DNA sequence data were processed and analyzed by Qiime (http://qiime.org/) and UPARSE (http://drive5.com/uparse/). Paired-end reads from the original DNA fragments were combined using FLASH18. All sequences were aligned with the SILVA bacterial 16S rRNA database. Sequences were clustered into operational taxonomic units (OTUs) at a cutoff of 97% sequence identity, after which the unweighted UniFrac in principal coordinate analysis (PCoA) was determined by Qiime. Some indices (rarefaction curves, Chao, Simpson, Shannon, coverage) were calculated to reveal Alpha diversity using mothur v.1.32. (http://www.mothur.org).

The Illumina MiSeq sequencing raw data have been deposited in the NCBI Sequence Read Archive database, and the SRA accession is SRP148831.

#### Biolog test

BIOLOG ECO Plates and high-throughput sequencing were used in this study to monitor microbial communities. High-throughput sequencing has helped investigators identify changes in the microbial structure and diversity (Alpana et al. 2017).

BIOLOG Eco-plates were used to investigate Sole-Carbon-Source Utilization (SCSU) of different microbial communities. Each plate contained a total of 31 carbon sources in triplicate. The suspension samples required a 5-min resting after they were obtained from the biofilter. Then, the supernatant was diluted to make the OD_600_ close to 0.05. Next, 150 µL aliquots of the diluted supernatants were added to the wells of the microplate and incubated at 30 °C for 3–4 days.

Data from the Biolog experiments were subsequently analyzed for metabolic function of microbial communities. The ability to degrade each of the 31 carbon source was indicated by average well color developments (AWCD):

$${\text{AWCD}} = \frac{{\sum_{{{\text{i = }}1}}^{96} {\left( {{\text{A}}_{\text{i}} - \frac{{{\text{A}}_{ 1} {\text{ + A}}_{ 3 3} {\text{ + A}}_{ 6 5} }}{3}} \right)} }}{93}.$$

In this equation, A_i_ refers to the OD_600_ value in well number “i”, among which No. 1, 33, and 66 were blank values.

Metabolic activities were indicated by changes in the AWCD rate (v) obtained in during the rapid growth rate as follows (Choi and Dobbs 1999):

$$v = \frac{{{\text{AWCD}}_{1} - {\text{AWCD}}_{0} }}{t},$$

where AWCD_0_ is the AWCD value at the beginning of the rapid growth period, AWCD_1_ is the AWCD value at the end of the rapid growth period and t is the length of the rapid growth period in hours.

The results were then subjected to principal component analysis (PCA) using SPSS 22.

## Results

### Removal and mineralization of VOCs

**Fig. 1:**
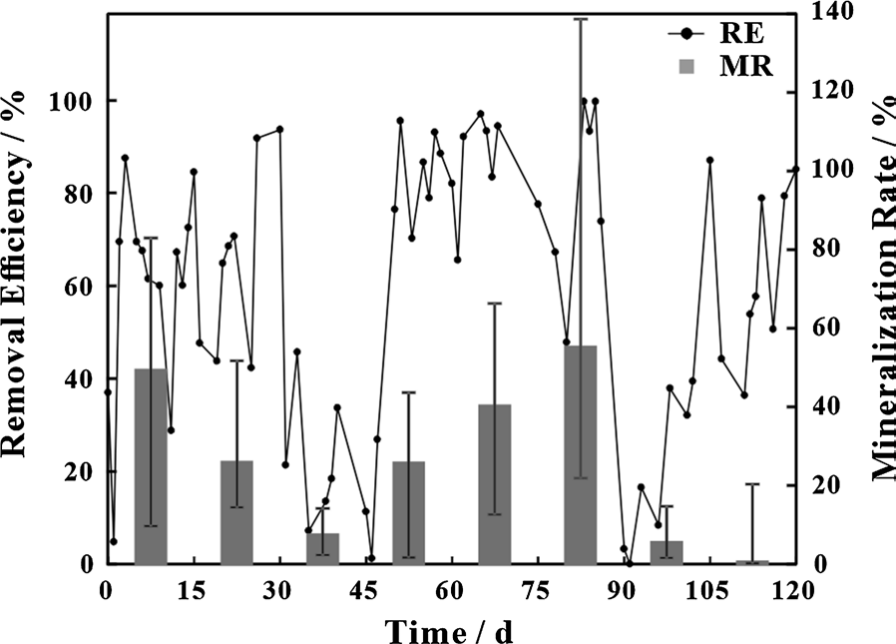
Removal efficiencies (RE) and average mineralization rates (MR) of the biofilter with shifting of VOCs (inlet VOCs were changed every 15 days with the order of toluene, ethylbenzene, chlorobenzene, acetone, isopropyl alcohol, ethyl acetate, n-hexane and tetrahydrofuran)

Shifting of inlet VOCs influenced removal efficiencies were observed (Fig. 1). Specifically, significant declines in removal efficiency were obtained after shifting VOCs; however, it recovered several days later, indicating that the system required time to adapt to the new conditions. Differences in molecular structures are known to impact the biodegradability of different compounds. For example, simpler compounds such as ethanol are more readily degraded by microorganisms than more complex materials (Sempere et al. 2008). In the present study, the removal efficiency varied from 18.2% (chlorobenzene) to 92.3% (isopropyl alcohol). The maximum elimination capacities of different volatile organic compounds are listed in Table 1. Alcohols, ketones, esters and ethers appeared to be more easily removed, whose eliminated capacities were higher than 600 mg m^−3^ min^−1^. Moreover, the system showed satisfactory performance for degrading toluene (678.11 mg m^−3^ min^−1^) and tetrahydrofuran (401.53 mg m^−3^ min^−1^). Conversely, chlorobenzene and n-hexane were not easily degraded by microorganisms, as indicated by removal efficiencies lower than 30%. Overall, considerable differences in degradation performance were seen among different VOCs.

**Table 1:** Performances of the biofilter with different inlet VOCs

VOCs	Inlet concentration (mg m^−3^)	Removal efficiency (%)	Elimination capacity (mg m^−3^ min^−1^)
Toluene	905	57.9	678.11
Ethylbenzene	655	67.8	574.70
Chlorobenzene	639	18.2	150.50
Acetone	598	83.7	647.74
Isopropyl alcohol	839	92.3	1002.16
Ethyl acetate	744	91.9	884.83
n-hexane	1009	29.5	385.20
Tetrahydrofuran	421	73.7	401.53

Similar results were observed among VOC mineralization rates (Fig. 1). Surprisingly, the mineralization rate of tetrahydrofuran was lowest among all tested compounds. More tetrahydrofuran was degraded into intermediates or microorganisms instead of CO_2_ and H_2_O.

Different performance was observed for different inlet VOCs, suggesting that the characteristics of the microbial community changed with inlet VOCs.

### Microbial diversity

The microbial community was analyzed following changes in VOCs (Table 2). The highest OTU value of 690 was observed for toluene, while all other VOCs had OTU values of less than 380. The ACE and Chao1 index values showed a similar trend, confirming that there was higher microbial diversity when treating toluene.

**Table 2:** Indexes of microbial following treatment with different types of VOCs

VOCs	OTU	ACE	Chao1	Coverage	Shannon	Simpson
Toluene	690	998.41	971.64	0.9959	4.73	0.9062
Ethylbenzene	357	487.79	459.24	0.9976	3.34	0.7778
Chlorobenzene	372	499.56	473.51	0.9969	3.28	0.7549
Acetone	357	414.83	395.65	0.9963	5.03	0.9322
Isopropyl alcohol	335	423.24	404.93	0.9976	4.39	0.8965
Ethyl acetate	357	404.22	392.05	0.9974	5.13	0.9248
N-hexane	285	364.87	359.59	0.9977	5.14	0.9481
Tetrahydrofuran	312	374.90	356.29	0.9976	5.06	0.9446

Shannon’s index was lower for compounds of aromatic hydrocarbons, indicating lower microbial diversity. A similar trend was observed for Simpson’s index, indicating that a higher microbial diversity led to lower dominance of the dominant species, as occurred with toluene. High consistency was seen between degrading performance and microbial community. As previously mentioned, higher microbial diversity was observed for toluene, which had the highest biodegradability among the aromatic hydrocarbons.

### Microbial community structure

**Fig. 2:**
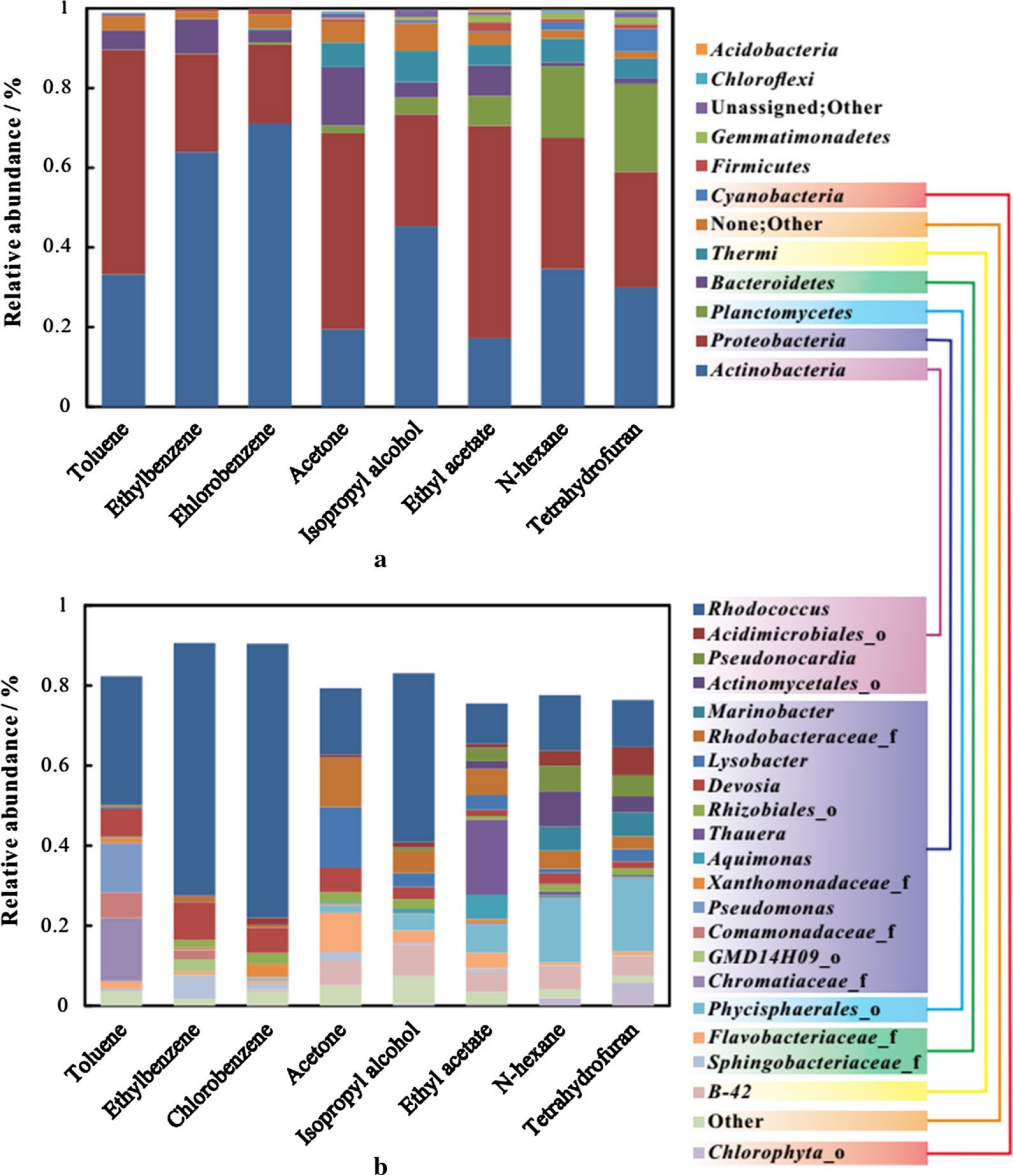
Figure 3-2 Microbial community structures (**a** relative abundances of microorganisms at the phylum level following the inlet of different volatile organic compounds; **b** relative abundances at the genus level when treating different volatile organic compounds. 22 genera were included, including the five genera with the highest abundance in each stage)

During the course of varying inlet VOCs, 21 phyla were found. Ten known phyla and two unidentified phyla were found to account for over 98% of the abundance (Fig. 2a).

*Actinobacteria* and *Proteobacteria* remained dominant throughout the experimental period. Upon further analysis, the abundance of *Actinobacteria* was found to fluctuate from 17 to 72%, while that of *Proteobacteria* varied from 19 to 57%. As the inlet VOCs were changed from toluene to ethylbenzene and chlorobenzene, the abundance of *Actinobacteria* increased in a stepwise fashion to 33.2%, 63.9% and 71.1%, respectively, while that of *Proteobacteria* decreased from 56.4 to 24.6% and then 19.8%. Surprisingly, the sum of the two phyla remains stable, with values of 89.6%, 88.5% and 90.9% being observed for toluene, ethylbenzene and chlorobenzene, respectively.

The changes in the microbial community occurred when the VOCs were switched from aromatic to non-aromatic hydrocarbons. The sum of *Actinobacteria* and *Proteobacteria* decreased from 90% to approximately 60%–70%, while the abundances of *Planctomycetes* and *Thermi* increased significantly after the hydrocarbons were changed. The abundance of the phylum *Planctomycetes* increased when some aromatic chemicals were removed. Moreover, their abundance increased when the aromatic hydrocarbons were replaced with non-aromatic chemicals such as acetone. *Thermi* remained stable during purification of non-aromatic hydrocarbons, fluctuating at levels of 5.0–8.0%. The abundance of *Bacteroidetes* peaked at 14.6% in response to acetone and then decreased to 1.0% upon application of *n*-hexane. These findings indicate that acetone was a better carbon and energy source for species in the phylum *Bacteroidetes* than n-hexane. The abundance of *Cyanobacteria* tripled in the presence of tetrahydrofuran when compared to other compounds. It has been speculated that members of the phylum *Cyanobacteria* could include more tetrahydrofuran degraders than other phyla.

A total of 264 genera were obtained during the operation, 21 of which were present in high abundance. The sum of these dominant genera accounted for 75%–91% of the total biomass (Fig. 2b). All genera belonged to the top seven phyla, with most belonging to the phyla *Actinobacteria* and *Proteobacteria*. Four genera, including *Rhodococcus*, *Pseudonocardia* and other two unidentified genera from the phylum *Actinobacteria*, had high abundances. Among them, *Rhodococcus* had significant advantages in abundance throughout the experimental period. The phylum *Proteobacteria*, which contained *Pseudomonas*, *Devosia*, *Aquimonas*, *Marinobacter* and eight other genera, showed high stability and uniformity with increased abundance when treating various contaminants, as opposed to genera belonging to the phylum *Actinobactria.*

The genus *Rhodococcus*, which belongs to the phylum *Actinobacteria*, was obtained in high abundance (> 10%) throughout the operation. Surprisingly, higher values of relative abundance were observed when aromatic hydrocarbons were being eliminated. Similarly, the abundance of *Devosia* sp. increased in response to the inlet of aromatic hydrocarbons. During the operation, specific degraders were identified. Specifically, high abundance (18.8%) of *Thauera* was observed in the presence of ethyl acetate, while less than 1% was observed in the presence of other VOCs. In addition, the genus *Pseudomonas* showed highest abundance during the inlet of toluene, while it comprised less than 0.5% in the presence of other VOCs.

### Microbial metabolic profile

**Fig. 3:**
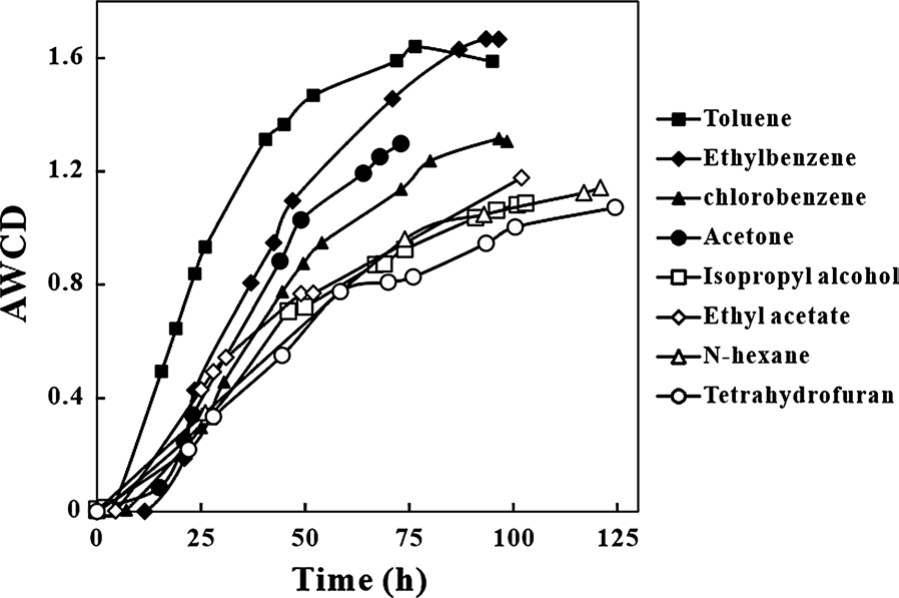
AWCD changes in microbial community under different inlet VOCs

The values of average well color development (hereafter AWCD, Fig. 3) were affected by density and activity of bacterial community (Preston-Mafham et al. 2002; Rutgers et al. 2016). The results showed that carbon source metabolic capacity of the microbial community decreased gradually with time. Initially, the AWCD values of toluene and ethylbenzene were higher than those observed for other VOCs. The rate of AWCD increase was 0.042 cm^−1^ h^−1^ for toluene and 0.035 cm^−1^ h^−1^ for ethylbenzene, which illustrated that the microbial community had higher activity in the presence of purified toluene than ethylbenzene. Growth rate declined to approximately 0.025 cm^−1^ h^−1^ when treating acetone and chlorobenzene and carbon source metabolic capacities and activities declined with time. The increase rate of AWCD when treating isopropyl alcohol, ethyl acetate, hexane and tetrahydrofuran were 0.013 cm^−1^ h^−1^, 0.014 cm^−1^ h^−1^, 0.010 cm^−1^ h^−1^ and 0.015 cm^−1^ h^−1^, respectively. Taken together, these results suggested that some measures might be taken to increase the activities of microorganisms after a long-term operation.

**Fig. 4:**
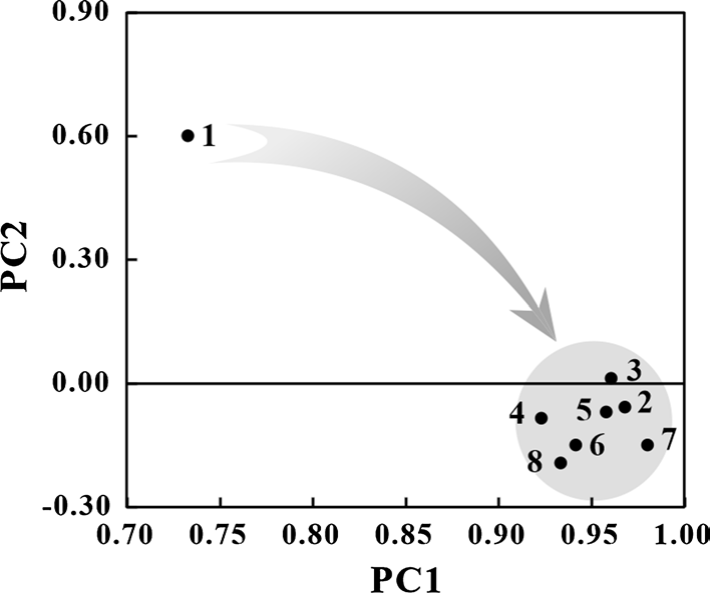
Carbon metabolism principle component analysis of microbial community with different inlet VOCs: No. 1–8 referred to stages of toluene, ethylbenzene, chlorobenzene, acetone, isopropyl alcohol, ethyl acetate, *n*-hexane and tetrahydrofuran

The duration between hours 20 and 30 was a period when the highest AWCD increasing rates were obtained. Therefore, principle component analysis was conducted in this study to obtain further insight (Fig. 4) based on data obtained between hours 20 and 30.

## Discussion

The dominant phyla *Actinobacteria* and *Proteobacteria* are the most commonly reported prokaryotic degraders (Coleman et al. 2006). As reported in previous studies, many members of the phylum *Actinobacteria* have aromatic hydrocarbon degrading abilities, and some species can utilize complicated chemicals as carbon or energy sources for growth; however, the capability for degradation decreases with increasing carbon chain length (Wen et al. 2014; Zylstra et al. 2000). *Proteobacteria* has previously been widely applied in bioremediation for hydrocarbon purification in combination with *Actinobacteria*, which is another dominant phylum commonly seen in aromatic hydrocarbon biodegradation (Fuentes et al. 2014). Hence, we can conclude that *Actinobacteria* and *Proteobacteria* played crucial roles in aromatic hydrocarbon purification in this study. Increasing abundance of the phylum *Planctomycetes* when some aromatic chemicals were being removed have also been confirmed in previous study (Delgado-Balbuena et al. 2016).

On genus level, it was previously reported that *Rhodococcus* can utilize and removal a large variety of pollutants (Maia et al. 2018; Warhurst and Fewson 1994). Members of the genus *Rhodococcus* were found to have the considerable ability to degrade a great number of aromatic hydrocarbons several decades ago (Sorkhoh et al. 1990). As well-known VOCs degraders, *Rhodococcus* species can utilize various chemicals and have therefore been applied frequently in VOCs purifications (Li et al. 2016). It was also confirmed that *Rhodococcus* species can degrade complicated chemicals like three to five rings polycyclic aromatic hydrocarbons (PAHs) (Song et al. 2011). Moreover, some strains of *Rhodococcus* were shown to have broad degradation capacities toward a mixture of 16 VOCs including benzene, toluene, ethylbenzene, *m*-xylene, *p*-xylene, *o*-xylene, and octane (Auffret et al. 2009). Because of its efficient and broad degradation capacities, *Rhodococcus* was used to remediate heavily PAH-contaminated soil with total PAHs of 375 mg, and up to 55% was removed (Sun et al. 2012). These performances could explain the high abundance of *Rhodococcus* throughout the experimental period and likely contributed greatly to the biodegradation performance in this system.

In consideration of other genera, the findings in this study are consistent with those of previous reports. High abundances of *Devosia* sp. were previously observed in aromatic compounds purification systems (Ramos et al. 2015), and they have been used as aromatic compounds degraders in previous studies (Papale et al. 2017). Ethyl acetate can be utilized as carbon resources by *Thauera* sp. (Du et al. 2017). The genus *Pseudomonas* was previously reported to be a toluene utilizer and degrader (Hernandez and Torre 2011; Su et al. 2014). *Pseudomonas* sp. was previously proposed to be indicators of biodegradation because of their sensitivity to substrate change (Obi et al. 2017; Yakimov et al. 2007).

The significant differences observed in this study indicated that target VOCs had significant effects on microorganisms, particularly at the genus level. The population of utilizers and degraders increased rapidly in the response to VOCs, with some becoming dominant species. Subsequently, changes in inlet VOCs caused dramatic decreases shifts in these populations. Taken together, these results indicate that the microbial community has the ability to adapt to new environmental conditions. Accordingly, there is the potential to develop specific measures to facilitate such adaptation to optimize biodegradation, such as increasing the abundance of specific degraders artificially.

The results revealed that the microbial community was significantly different when purifying toluene then when treating other VOCs. Surprisingly, the communities were highly similar in the presence of all other test compounds. This might have occurred because of significant microbial community shifts in the initial operations (Qiu et al. 2013). After the initial operation, the system became relatively stable, possibly indicating that the community stabilized with time.

Biodegradability of different VOCs has remarkable influences on microbial elimination performance, community structures and metabolic profiles. The highest diversity was obtained at the beginning of the operation while eliminating toluene. Diversity declined with prolonged operation, and significant differences were found in microbial community structures at both the phylum and genus levels. As the experiment continued, the carbon source metabolic capacity declined gradually. The metabolic characteristics of carbon source utilization differed significantly following toluene input, while it was similar among all other treatment groups. Artificial addition of degraders and measures to increase microbial activity might optimize biofiltration.

## Additional file

**Additional file 1: Figure S1.** Biofilter structure and the flow directions of air/water flow. **Table S1.** The characteristics of the selected VOCs.
