# Supplementary material for: Response of microbial community structure and metabolic profile to shifts of inlet VOCs in a gas-phase biofilter
Source: AMB Express. 2018 Oct 3;8:160. doi: 10.1186/s13568-018-0687-z (PMC6170518; doi:10.1186/s13568-018-0687-z)
Supplement: Supplementary file 1 — Additional file 1: Figure S1. Biofilter structure and the flow directions of air/water flow. Table S1. The characteristics of the selected VOCs. [file 13568_2018_687_MOESM1_ESM.doc]

**Additional File**

**Figure S1** Biofilter structure and the flow directions of air/water flow.

**Table S1** The characteristics of the selected VOCs.


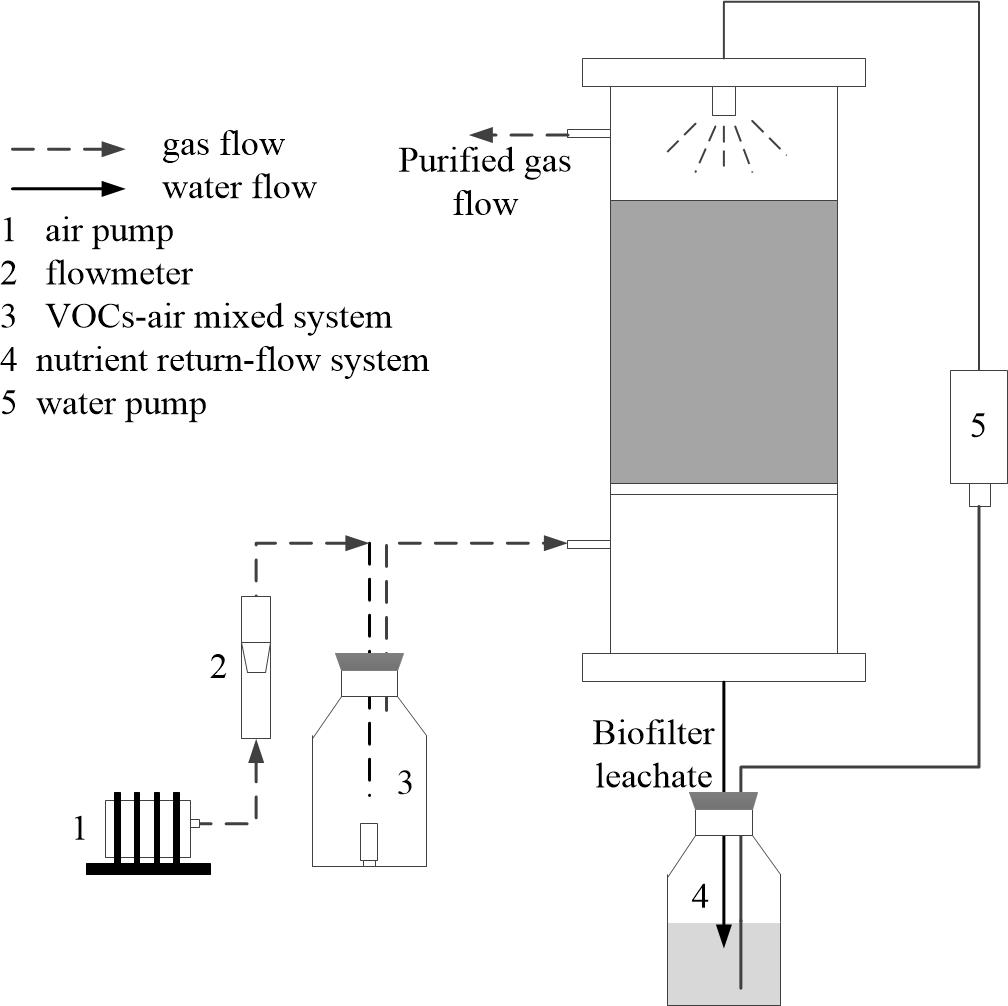


**Figure S1**

**Table S1**

| VOCs | Molecular  formula | Molecular  weight | Boiling  point/℃ | Henry's Law constant/ mol∙(kg∙bar)^-1^ |
| --- | --- | --- | --- | --- |
| Toluene | C_7_H_8_ | 92.14 | 111 | 0.15 |
| Ethylbenzene | C_8_H_10_ | 106.17 | 136 | 0.12 |
| Chlorobenzene | C_6_H_5_Cl | 112.56 | 132 | 0.27 |
| Acetone | C_3_H_6_O | 58.08 | 56 | 30 |
| Isopropyl alcohol | C_3_H_8_O | 60.10 | 82 | 88 |
| Ethyl acetate | C_4_H_8_O_2_ | 88.11 | 76.5-77.5 | 8.9 |
| N-hexane | C_6_H_14_ | 86.18 | 68.95 | 0.00076 |
| Tetrahydrofuran | C_4_H_8_O | 72.11 | 66 | 14 |
